# Exploring the measurement properties of the osteopathy clinical teaching questionnaire using Rasch analysis

**DOI:** 10.1186/s12998-018-0182-2

**Published:** 2018-05-03

**Authors:** Brett Vaughan

**Affiliations:** College of Health & Biomedicine, Victoria University, Melbourne, Australia

**Keywords:** Item response theory, Reliability estimation, Medical education, Clinical education

## Abstract

**Background:**

Clinical teaching evaluations are common in health profession education programs to ensure students are receiving a quality clinical education experience. Questionnaires students use to evaluate their clinical teachers have been developed in professions such as medicine and nursing. The development of a questionnaire that is specifically for the osteopathy on-campus, student-led clinic environment is warranted. Previous work developed the 30-item Osteopathy Clinical Teaching Questionnaire. The current study utilised Rasch analysis to investigate the construct validity of the Osteopathy Clinical Teaching Questionnaire and provide evidence for the validity argument through fit to the Rasch model.

**Methods:**

Senior osteopathy students at four institutions in Australia, New Zealand and the United Kingdom rated their clinical teachers using the Osteopathy Clinical Teaching Questionnaire. Three hundred and ninety-nine valid responses were received and the data were evaluated for fit to the Rasch model. Reliability estimations (Cronbach’s alpha and McDonald’s omega) were also evaluated for the final model.

**Results:**

The initial analysis demonstrated the data did not fit the Rasch model. Accordingly, modifications to the questionnaire were made including removing items, removing person responses, and rescoring one item. The final model contained 12 items and fit to the Rasch model was adequate. Support for unidimensionality was demonstrated through both the Principal Components Analysis/t-test, and the Cronbach’s alpha and McDonald’s omega reliability estimates. Analysis of the questionnaire using McDonald’s omega hierarchical supported a general factor (quality of clinical teaching in osteopathy).

**Conclusion:**

The evidence for unidimensionality and the presence of a general factor support the calculation of a total score for the questionnaire as a sufficient statistic. Further work is now required to investigate the reliability of the 12-item Osteopathy Clinical Teaching Questionnaire to provide evidence for the validity argument.

**Electronic supplementary material:**

The online version of this article (10.1186/s12998-018-0182-2) contains supplementary material, which is available to authorized users.

## Background

Clinical teaching influences the development of clinical and patient management skills students need for competent, safe and effective practice. At present, little is known about clinical education in osteopathy in non-United States teaching programs beyond the commentary on one Australian osteopathy program by Vaughan et al. [1]. These authors postulated that Collins’ cognitive apprenticeship model [2, 3] could account for a number of aspects of the student-clinical teacher interaction within a student-led clinical environment.

Osteopathy students undertaking their clinical education in Australia, New Zealand and United Kingdom are in their final years of training and are responsible for the management of patients under the supervision of a qualified osteopath (‘clinical teacher’). Clinical education in osteopathy is typically undertaken in a student-led, on-campus clinic environment – in the Australian context. Allan et al. [4] referred to these as ‘university clinics’. These clinics provide students with an opportunity to develop their work-readiness, and practice the application of skills and knowledge acquired in the classroom in a supervised environment. In osteopathy clinical education, each clinical teacher typically supervises between 5 and 7 students at any one time [1] however this may be up to 10 students in some instances [5]. The evaluation of this teaching is important to ensure students receive appropriate clinical skills education and development.

Systematic reviews of questionnaires to evaluate clinical teaching have been undertaken [6, 7]. These reviews have identified a substantial number of questionnaires with varying degrees of evidence of their validity or reliability. The statistical approaches to the development of these questionnaires are also variable. Both systematic reviews reported ‘factor analysis’ was used in the development of many of the questionnaires. It was not clear what methods were employed in all instances however it appears that Principal Components Analysis (PCA) was typically used. This was potentially due to convenience [8] (e.g. PCA is the default analysis in SPSS), or following how other researchers have developed clinical teaching evaluations [8], or a genuine desire to retain explained variance. Whilst PCA can be an effective approach to retain the least number of items to explain a substantial portion of the variance [9–12], the models produced often do not fit those generated by more advanced statistical techniques [13]. Extraction methods such as Principal Axis Factoring and ordinary (unweighted) least squares (OLS) are more appropriate than PCA [11], the latter (OLS) being particularly suitable for ordinal data that is typical of self-report questionnaires. In the last five years, researchers have used these Exploratory Factor Analysis (EFA) and Confirmatory Factor Analysis (CFA) approaches in the development of questionnaires to evaluate the quality of clinical teaching [14–16].

Previous work has developed the Osteopathy Clinical Teaching Questionnaire (OCTQ) [16]. The OCTQ is a 33-item questionnaire (30 items and 3 global rating items) designed to evaluate the quality of clinical teaching in on-campus, student-led osteopathy teaching clinics. Work undertaken thus far has provided evidence for the validity argument for the OCTQ through item development and EFA. The use of modern test theory [17] approaches for questionnaire development is particularly relevant in health sciences education and clinical research [18–21], where there is a desire to measure attitudes and abilities. The current study is the second to employ Rasch analysis in the ongoing development of a clinical teaching quality questionnaire, the other being that by Winstanley and White [22] in a revision of the Manchester Clinical Supervision Scale (MCSS). The aim of the current study was to explore the construct validity of the OCTQ by using Rasch analysis. Consistent with Kane’s approach to the development of a validity argument [23], the present study also aims to provide further evidence for the validity of the scores derived from the OCTQ.

## Methods

The study received ethics approval from Victoria University (VU) (Australia), Southern Cross University (SCU) (Australia), Unitec Institute of Technology (New Zealand), and the British School of Osteopathy (BSO) (United Kingdom). Participation in the study was voluntary and did not impact on the ability of the students to receive their grades nor graduate from their program of study. Results from this study were not used for employment or promotion decisions nor made available to the clinical teachers’ supervisor, however copies of the anonymous student responses were provided to the individual clinical teacher upon request. Consent to participate in the study was implied upon the return of a completed questionnaire.

### Participants

Students in the final two years of the programs at VU, SCU, Unitec, and the BSO were invited to participate in the study. These students were completing the clinical practice requirement of their respective programs and were at similar stages in their clinical training. Students received an email via their university email address. They were informed of the study and encouraged to complete a questionnaire for each of the clinical teachers whom they had worked with in the period July 2014 – December 2014 (VU and SCU), and March 2015 – July 2015 (Unitec and BSO). The clinical teachers at each institution also received an email informing them the study was taking place. Students were not required to identify themselves on the questionnaire.

### Data collection

Students completed version 2 of the Osteopathy Clinical Teaching Questionnaire (OCTQ) [16] (Additional file 1) during their scheduled clinic placement time. The OCTQ (version 2) is a 33-item questionnaire that contains 30 items evaluating different aspects of the clinical teachers’ performance across 5 factors: learning environment; modelling; feedback; patient management; and reflective practice. There are also 3 global rating items (Additional file 1). Each item is anchored with the statement “This Clinical Educator…” and rated on a scale of 1 (strongly disagree) to 5 (strongly agree) with a neutral category (option 3). The students were asked to complete the OCTQ (version 2) for each of the clinical teachers they had worked with, basing their responses on the entirety of their interaction with the teacher for the relevant teaching period and not focusing on a single positive and/or negative encounter with the teacher. Responses were anonymous – neither the student nor clinical teacher being rated were identified. The student was asked to indicate their gender and the gender of the clinical teacher being rated as previous research has identified that student and teacher gender can influence responses to clinical teaching questionnaire items [24]. The institution where the questionnaire was completed was also noted.

### Data analysis

Descriptive statistics were generated in *R* [25] using the *psych* package [26].

#### Rasch analysis

**Fig. 1:**
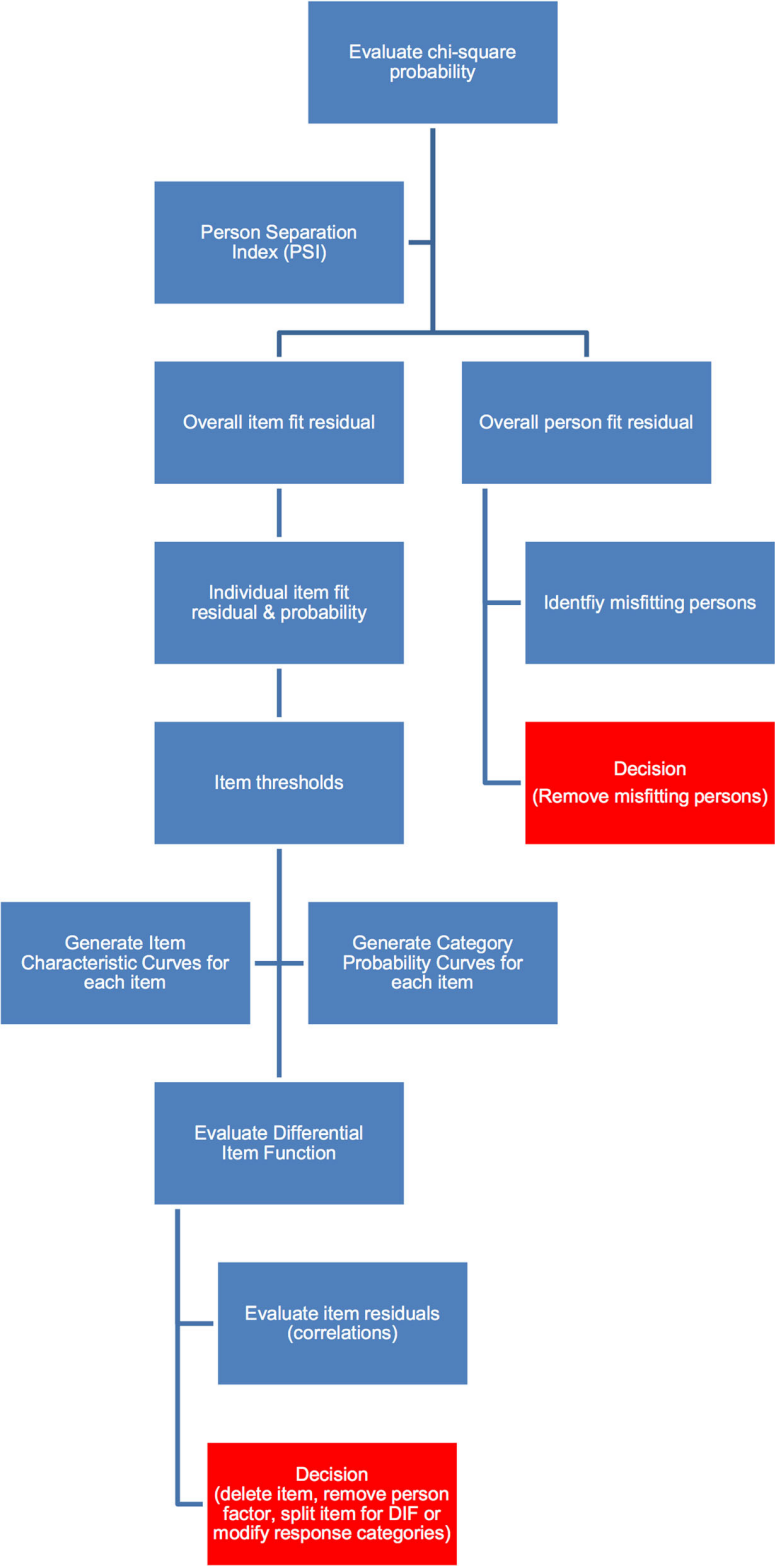
Flow diagram outlining the statistics used in each step of the Rasch analysis. At the red box, the process is repeated until fit to the Rasch model is achieved

Data were entered into Microsoft Excel for Mac then exported to RUMM2030 [27] for Rasch analysis (RA). The target construct in the present study is the quality of clinical teaching provided by osteopathy clinical teachers. As each OCTQ (version 2) item was scored on a 1–5 scale the polytomous Rasch model was used for the analysis. Each step in the RA informed the next. Within each step, a number of statistical analyses were undertaken to determine the most appropriate action for the next step. Figure 1 presents the analyses undertaken within each step.

#### Rasch model fit

Overall model fit was first evaluated using the chi-square statistic and Bonferonni-adjusted *p*-value. Fit residual standard deviations (SD) were then used to evaluate the fit of the items and persons respectively to the Rasch model [18] along with Bonferonni-adjusted chi-square probabilities. The Person Separation Index (PSI) was calculated at each step. Differential item function was then evaluated for each item using the person factors institution, clinical educator gender and student gender. Person fit was evaluated using the fit residual statistic, with responses from misfitting persons removed from subsequent steps. Correlations between each of the items were evaluated to identify item combinations with residuals greater than 0.20 suggesting ‘local dependence’ [28]. To determine if the local dependence was impacting on the PSI, a subtest analysis was performed in RUMM2030. A reduction in the PSI with the subtest suggests the item combination is inflating this value and requires the removal of one of the items. The information from each of these analyses informed the next step (e.g. remove an item/person response, rescore an item). Once fit to the Rasch model was achieved, the PSI informed the number of possible strata that could be identified [29]. Given clinical teaching evaluation data are unlikely to be normally distributed [7], the method described by Wright [30] to identify each strata was used. This method included the addition of 10% to the standard error for each logit “…to allow for the unmodeled noise encountered in real data” (p. 786).

#### Dimensionality testing, reliability estimates and descriptive statistics

Multiple approaches were employed to evaluate the dimensionality of the questionnaire to ascertain whether the items were measuring the same latent construct. These approaches were PCA of the standardised residuals, and evaluating the number of factors to extract using methods for EFA.

#### Principal components analysis

Once a fit to the Rasch model was achieved through each of the analyses, a PCA of the standardised residuals was undertaken to derive the ‘Rasch factor’ or ‘Rasch dimension’. An independent t-test was used to evaluate the difference between the items that loaded positively and negatively onto the ‘Rasch factor’. The binomial confidence interval for the t-test was calculated in R [25] using the binom package [31].

Number of factors to extract Parallel analysis (PA) [32], eigenvalues, acceleration factor (AF) [33] and optimal coordinates (OC) [33] were the methods used to confirm the number of factors to extract. These procedures were performed using the *psych* (version 1.5.4) [26] and *nFactors* (version 2.3.3) [34] packages in *R* utilising the polychoric correlation generated using the *polycor* package (version 0.7–8) [35].

#### Reliability estimates

Three reliability estimates were calculated using a variety of statistics in the *psych* package [26] in *R* [25]: Cronbach’s alpha (α); and McDonald’s omega hierarchical (ω_h_) and total (ω_t_) [36–38]. High ω_h_ values suggest that general factor accounts for the total score variance supporting unidimensionality [39], and values greater than 0.5 have been suggested to support the calculation of a total score for all scale items [40]. Omega subscale (ω_s_) was also calculated for the subfactors identified when calculating the ω coefficient. Each of the reliability estimates were calculated using the polychoric correlation given the underlying data were ordinal in nature [41, 42], and also calculated based on the raw data. The explained common variance (ECV) was also calculated to further evaluate unidimensionality. Higher ECV values support unidimensionality [39, 43] however there is no guidance as to an acceptable value [39].

## Results

Four hundred questionnaires were received. One questionnaire was not completed therefore 399 were available for analysis. Demographic data are presented in Table 1.

**Table 1:** Demographic data

			Institution			
		Total	Victoria University	Southern Cross University	British School of Osteopathy	Unitec
Total responses		399	149	119	42	89
Student gender	Male	150	44	58	14	34
	Female	224	98	49	26	51
Clinical Educator gender	Male	261	78	108	31	44
	Female	123	65	9	8	41

Note: some participants did not indicate either their gender or the gender of the clinical educator being rated

The category response frequencies were negatively skewed, however responses were observed for each item across the five response categories. The neutral *Neither agree nor disagree* response category was used, on average, 12% of the time suggesting the usefulness of this category.

### Rasch analysis

#### Overall Rasch model fit

**Fig. 2:**
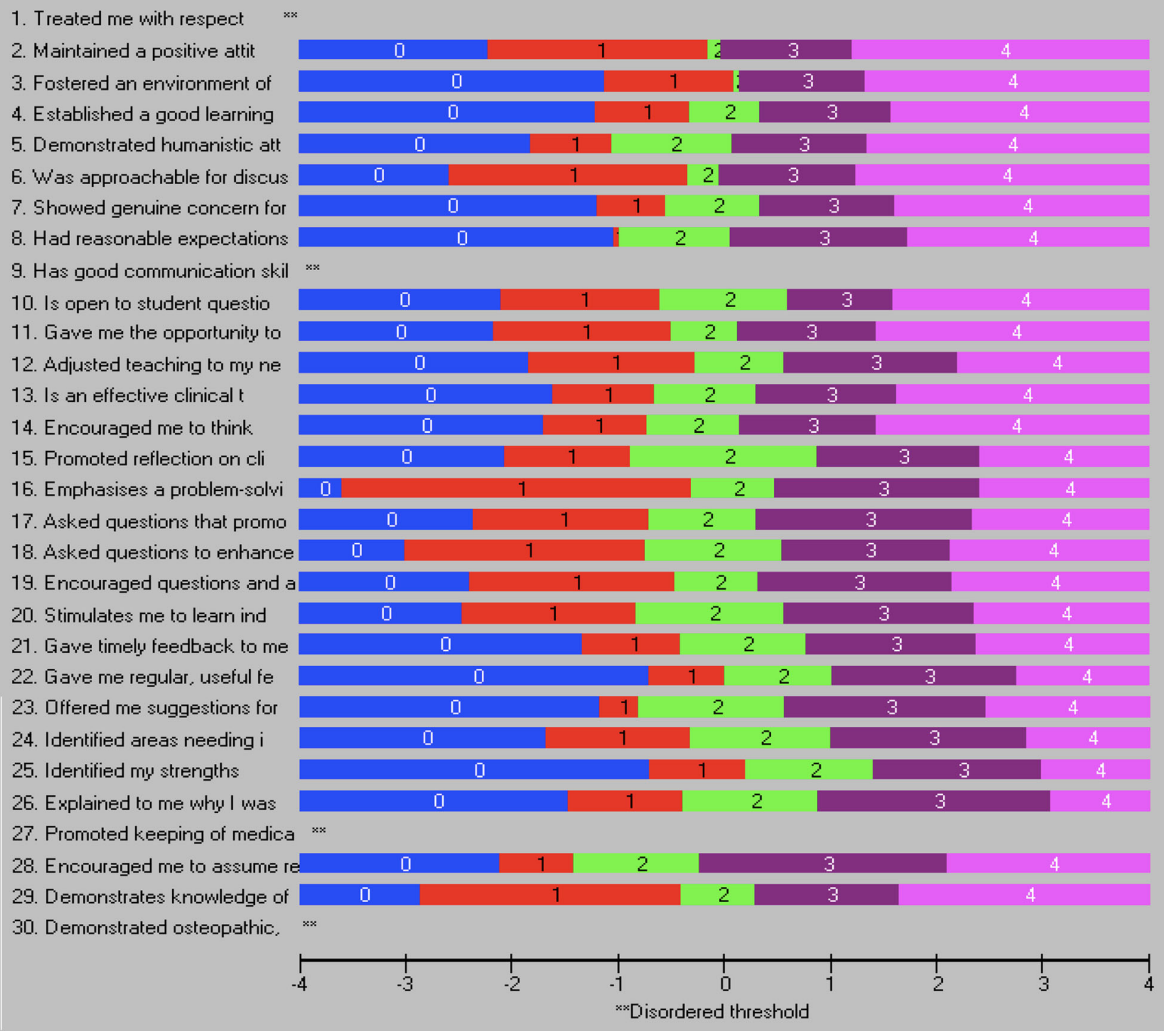
Threshold map for the Osteopathy Clinical Teaching Questionnaire (version 2) items

The Likelihood ratio test was statistically significant (*p* < 0.001), subsequently the partial credit model was used for the Rasch analysis. Overall fit was significantly different to the Rasch model (χ^2^(150, *N* = 399) = 407.42, *p* < 0.001) with a PSI of 0.910, item fit residual mean of − 0.32 (SD 2.34) and person fit residual mean of − 0.57 (SD 2.03). Item fit statistics and the threshold map for the initial analysis are presented in Table 2 and Fig. 2 respectively. Sixty responses were identified as *extreme* by RUMM and a further 62 responses demonstrated person fit residual SDs of greater than 2.5 suggesting misfit to the Rasch model.

**Table 2:** Item fit statistics for the full 30-item Osteopathy Clinical Teaching Questionnaire

Item	Location	Fit Residual	χ^2^	Probability
1	−0.338	−2.592^a^	9.220	0.101
2	−0.292	−1.144	3.301	0.658
3	0.115	−2.670^a^	9.115	0.104
4	0.104	−2.747^a^	11.628	0.040
5	−0.360	−0.501	5.967	0.309
6	−0.426	−1.513	7.659	0.176
7	0.063	−1.644	4.684	0.457
8	−0.056	1.023	5.296	0.381
9	−0.129	−1.866	7.391	0.193
10	−0.117	0.085	3.599	0.608
11	−0.272	−2.476	10.194	0.070
12	0.167	−1.909	10.186	0.070
13	−0.079	−2.155	17.007	0.005
14	−0.205	−1.581	4.161	0.526
15	0.092	1.082	4.790	0.442
16	−0.244	−1.347	2.679	0.749
17	−0.101	−2.614^a^	13.235	0.023
18	−0.263	−1.744	7.138	0.211
19	−0.091	−2.881^a^	14.378	0.014
20	−0.090	0.885	5.992	0.307
21	0.358	0.662	7.751	0.170
22	0.778	0.357	10.095	0.072
23	0.268	−0.109	8.925	0.112
24	0.467	4.217^a^	43.774	0.000^b^
25	0.978	3.859^a^	35.118	0.000^b^
26	0.529	1.770	15.103	0.009
27	0.395	7.145^a^	105.105	0.000^b^
28	−0.412	0.477	2.452	0.783
29	−0.327	1.384	15.011	0.010
30	−0.511	−1.280	6.476	0.262

^a^Item fit residual greater than 2.5. Large negative residuals suggest item redundancy, large positive residuals

^b^Statistically significant chi-square probability (Bonferonni-adjusted *p* = 0.0003)

### Modifications to the model

Extreme and misfitting responses (*n* = 122) were removed. Once completed the overall fit remained significantly different to that of the Rasch model (χ^2^(150, *N* = 277) = 315.98, *p* < 0.001), the PSI (0.927), however the item fit residual mean − 0.34 (SD 2.09) and person fit residual mean − 0.27 (SD 1.11) all improved. Local dependence was observed for a number of items (Additional file 2). Two iterations of the analysis were undertaken to produce a fit to the Rasch model. The steps to produce this model are at Additional file 3 and the fit statistics were reviewed after each modification in order to determine the next step in the analysis.

**Fig. 3:**
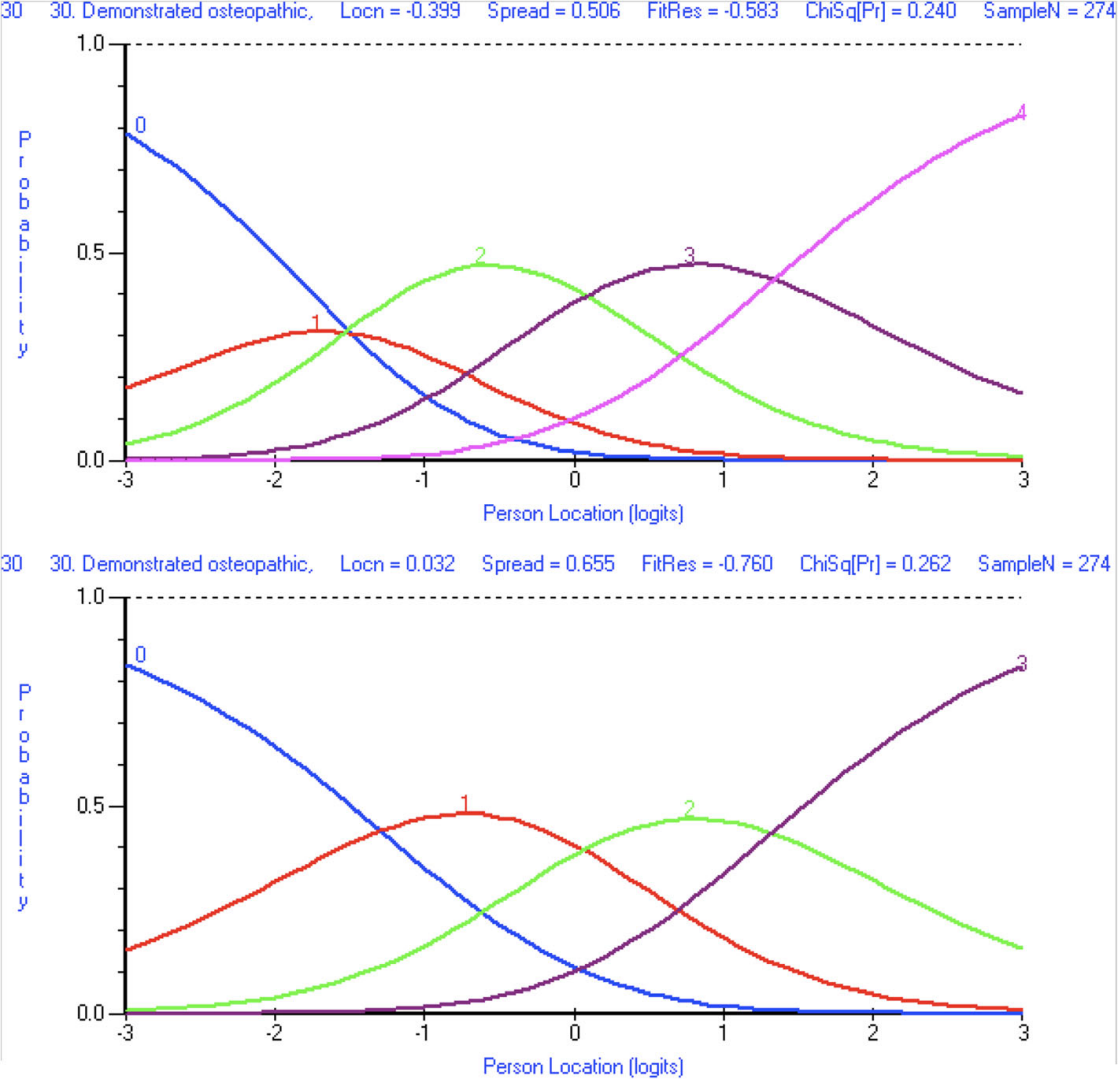
Category probability curve for Osteopathy Clinical Teaching Questionnaire item 30 *Demonstrated osteopathic, clinical examination and rehabilitation knowledge and skill(s)*. Before item rescoring (top image). After item rescoring (bottom image)

Osteopathy Clinical Teaching Questionnaire (OCTQ) items were removed where they demonstrated misfit to the Rasch model, differential item functioning, or local dependence. Additional file 3 identifies the reasons for the removal of the items during the analysis. Item 30 *Demonstrated osteopathic, clinical examination and rehabilitation knowledge and skill(s)* required rescoring in order to improve its fit to the Rasch model, as respondents did not appear to be using the *Strongly Disagree* and *Disagree* responses in the manner predicted by the Rasch model. Figure 3 demonstrate the category probability curves pre- and post-rescoring.

#### Differential item function analysis

**Fig. 4:**
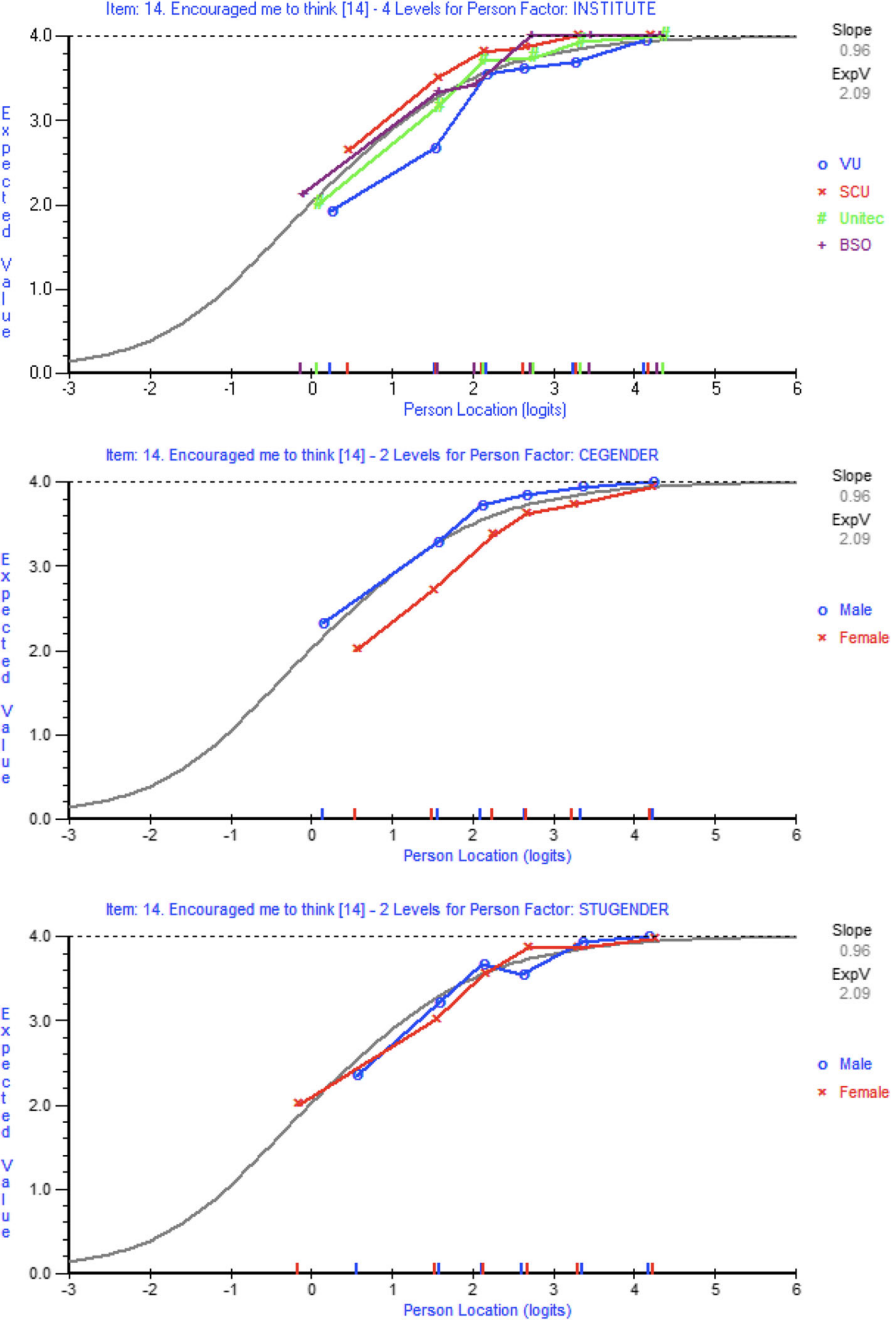
Item characteristic curves for Osteopathy Clinical Teaching Questionnaire item 14 *Encouraged me to think.* Differential item function for institution (top image). Differential item function for clinical teacher gender (middle image). Differential item function for student gender (bottom image)

Uniform differential item function (DIF) was observed for institution (Fig. 4), clinical teacher gender (Fig. 4), and student gender (Fig. 4) for item 14 *Encouraged me to think*. For gender, female clinical teachers received scores that were systematically lower than that expected by the Rasch model, and were significantly lower when compared to males across all of the class intervals. With regard to student gender, systematic differences existed between males and females across the class intervals however whether males or females selected higher responses was not consistent.

Other items that demonstrated DIF through each iteration included item 19 *Encouraged questions and active participation* and item 28 *Encouraged me to assume responsibility for patient care*. In order to ensure that the items on the modified OCTQ were applicable to a range of teaching institutions and free from gender influence, any item demonstrating DIF was removed. Those items demonstrating local dependence were analysed using a subtest to examine whether they were inflating the PSI value, and one item removed (Additional file 3).

#### Final Rasch model

**Fig. 5:**
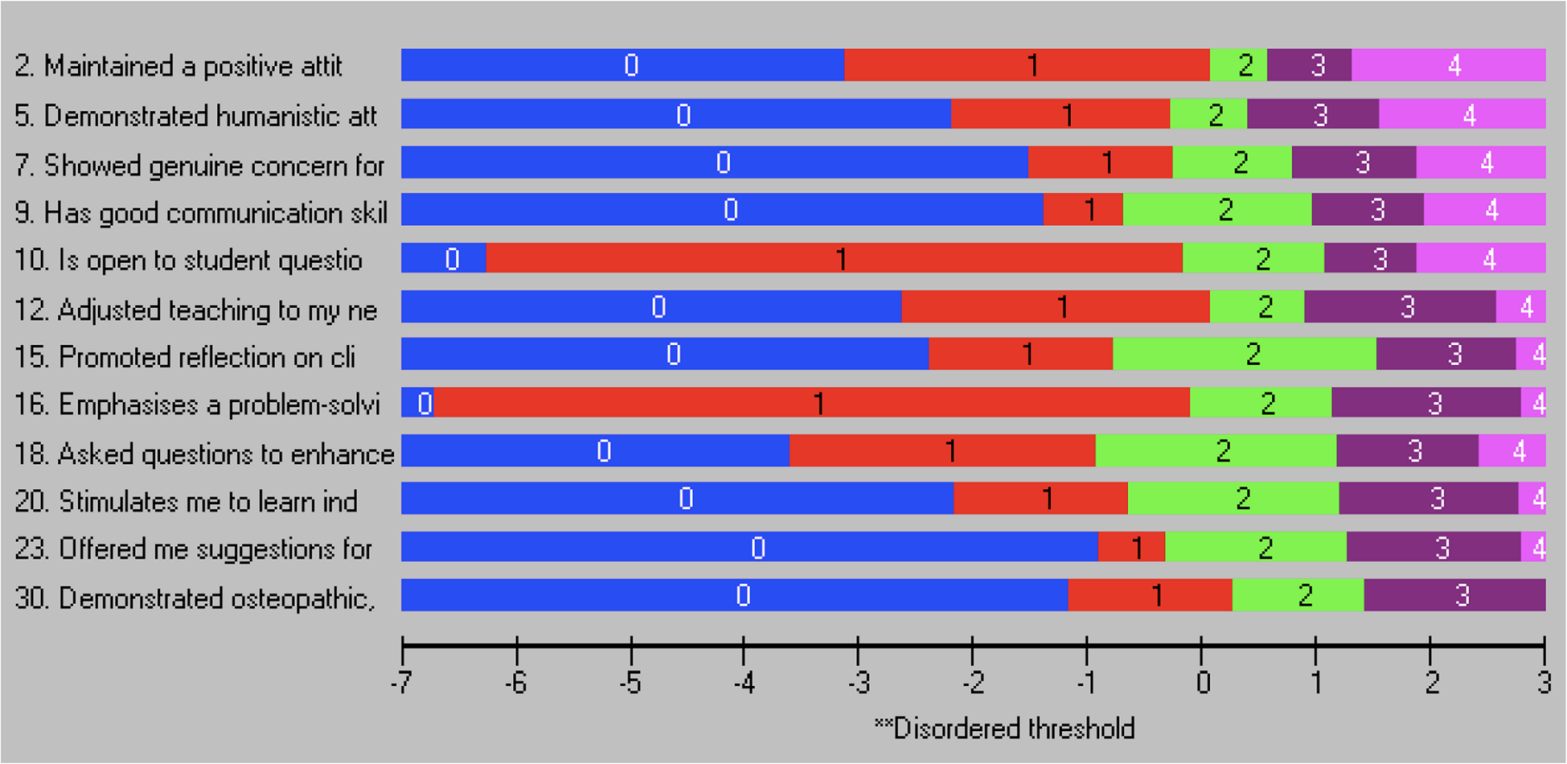
Threshold map for the 12-item Osteopathy Clinical Teaching Questionnaire

Fit to the Rasch model was achieved by removing 18 items, rescoring item 30, and removing misfitting 153 responses in total comprising 122 at the initial analysis and 31 misfitting responses identified during the subsequent analysis (Additional file 3 and Additional file 4). The final model contained 12 items and 246 responses. Overall fit to the Rasch model was demonstrated (χ^2^(60, *N* = 246) = 65.26, *p* = 0.298). The item and person fit residual means were − 0.34 (SD 1.18) and − 0.20 (SD 0.82). These fit residual SDs were both within the acceptable range. The item fit statistics are presented in Table 3 and the threshold map at Fig. 5. There is a spread of item location values that represent different levels of a single latent construct (Table 3).

**Table 3:** Item fit statistics for the 12-item Osteopathy Clinical Teaching Questionnaire

Item	Location	SE	Fit Residual	χ^2^	Probability
*This Clinical Educator:*					
2. Maintained a positive attitude towards me	− 0.283	0.104	0.311	1.293	0.935
5. Demonstrated humanistic attitudes in relating to patients (integrity, compassion and respect)	−0.118	0.106	−1.559	4.633	0.462
7. Showed genuine concern for my professional well-being	0.232	0.099	−0.816	4.057	0.541
9. Has good communication skills	0.225	0.099	−1.479	7.065	0.216
10. Is open to student questions and alternative approaches to patient management	−0.856	0.098	−0.391	6.938	0.225
12. Adjusted teaching to my needs (experience, competence, interest)	0.236	0.096	−1.675	9.890	0.078
15. Promoted reflection on clinical practice	0.292	0.096	1.073	2.665	0.751
16. Emphasises a problem-solving approach rather than solutions	−0.714	0.097	−1.589	3.700	0.593
18. Asked questions to enhance my learning	−0.224	0.100	−0.917	12.669	0.027
20. Stimulates me to learn independently	0.304	0.097	0.415	3.068	0.689
23. Offered me suggestions for improvement when required	0.723	0.093	1.900	4.945	0.423
30. Demonstrated osteopathic, clinical examination and rehabilitation knowledge and skill(s)	0.183	0.117	0.591	4.339	0.501

*SE* Standard error

**Fig. 6:**
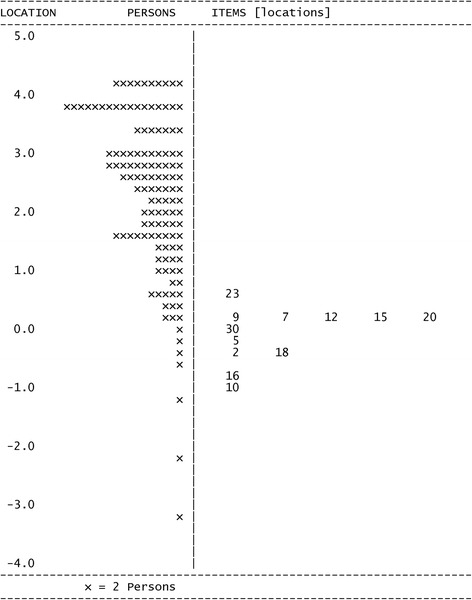
Person-item distribution (Wright map) for the 12-item Osteopathy Clinical Teaching Questionnaire

**Fig. 7:**
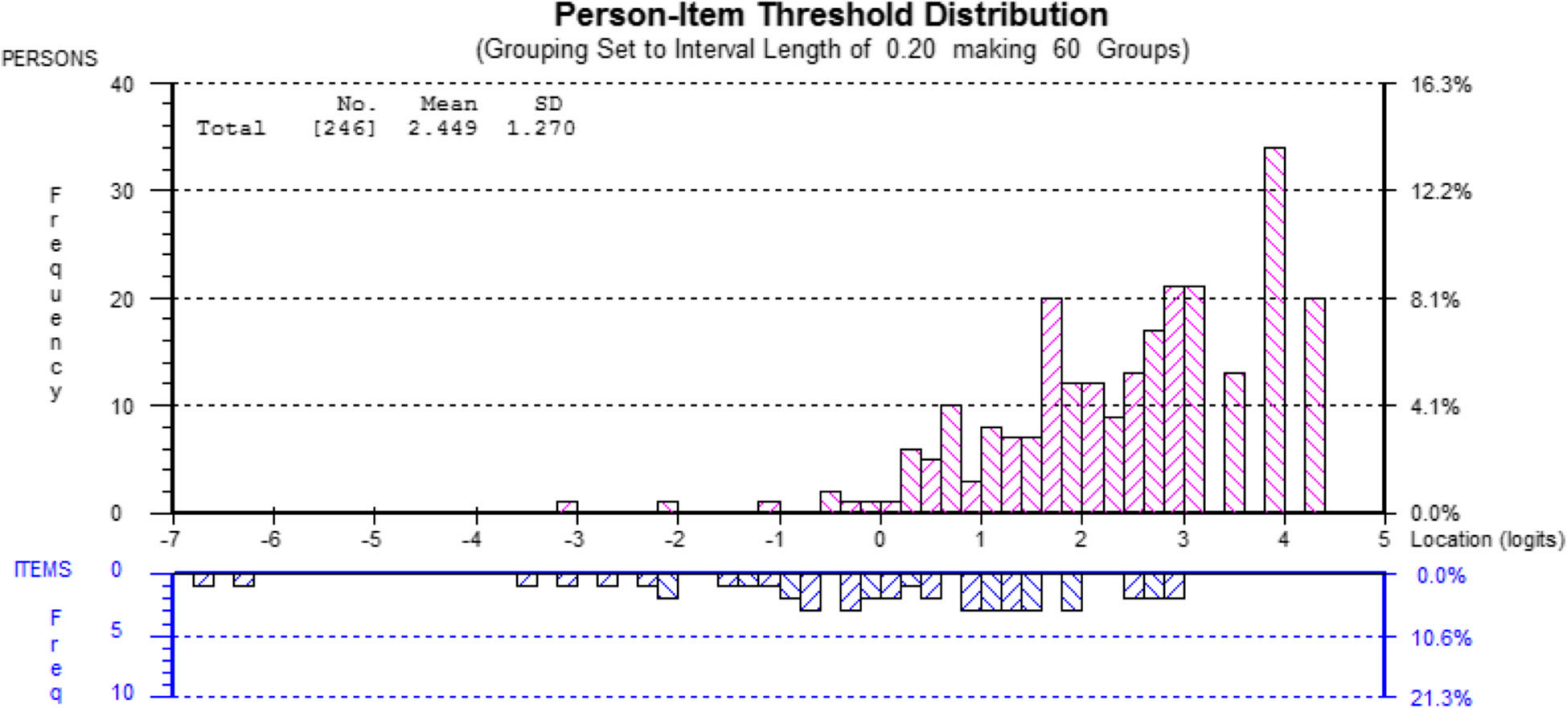
Person-item threshold distribution for the 12-item Osteopathy Clinical Teaching Questionnaire

The mean person location logit was 2.44 suggesting that respondents used the higher options of the 1–5 scale for each OCTQ item. The item-person map and the item-threshold distribution are shown in Figs. 6 and 7 respectively. The item-threshold distribution shows that the OCTQ item scale covers a range of possible options on the 1–5 scale, and largely covers the responses provided in the present study. The questionnaire may be subject to a ‘ceiling-effect’ however.

No item in the final model demonstrated DIF or local dependence. The PSI was 0.827 suggesting that approximately 83% of the variance in the observed score on the final 12-item questionnaire is due to the true variance in a students’ perceived quality of teaching provided by a clinical teacher. The remaining 17% is classified as error variance. The PSI also indicates that 3–4 strata could be identified [29].

The ‘Rasch factor’ or first component of the PCA accounted for 17.13% of the variance, suggesting the questionnaire is unidimensional. The PCA/t-test identified twelve responses where the t-tests were significantly different between the OCTQ items that loaded positively and negatively (Table 4) onto the ‘Rasch factor’ (*p* = 0.04, 95% CI 0.021–0.085). As the 95%CI (calculated using the 11 statistics in the *binom R* package) contains the value of *p* = 0.05, this provides further evidence to support the unidimensionality of the 12-item questionnaire.

**Table 4:** Principal Component Analysis of the residuals for the 12-item Osteopathy Clinical Teaching Questionnaire

Item	Loading on ‘Rasch factor’
2. Maintained a positive attitude towards me	**0.515**
5. Demonstrated humanistic attitudes in relating to patients (integrity, compassion and respect)	**0.396**
7. Showed genuine concern for my professional well-being	0.172
9. Has good communication skills	**0.538**
10. Is open to student questions and alternative approaches to patient management	**0.504**
12. Adjusted teaching to my needs (experience, competence, interest)	**0.351**
15. Promoted reflection on clinical practice	**−0.538**
16. Emphasises a problem-solving approach rather than solutions	**−0.508**
18. Asked questions to enhance my learning	**−0.464**
20. Stimulates me to learn independently	**−0.333**
23. Offered me suggestions for improvement when required	−0.242
30. Demonstrated osteopathic, clinical examination and rehabilitation knowledge and skill(s)	−0.066

Items in bold were used in the t-test

### Factor extraction

**Fig. 8:**
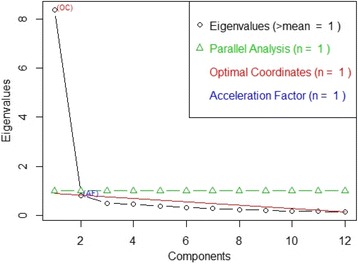
Number of factors to extract for the 12-item Osteopathy Clinical Teaching Questionnaire

Additional support for the unidimensionality of the 12-item questionnaire was obtained from the four methods used in EFA to determine the number of factors to extract. Using the data from the 399 completed questionnaires and the subsequent polychoric correlation of this data, all four methods suggested one factor should be extracted (Fig. 8). All valid responses were used in this analysis to ensure the accuracy of the result.

### Reliability estimates

Reliability estimates for the 12-item OCTQ (Table 5) using the polychoric correlation were slightly larger compared to the raw data, consistent with Revelle’s [44] suggestion that ω_h_ can be overestimated with polychoric correlations. All of the reliability estimates, regardless of whether the raw data or polychoric correlation was used, were well above an acceptable level of 0.80 suggesting less than 20% of the variance is measurement error. This level of measurement error is consistent with the PSI.

**Table 5:** Reliability estimates for the 12-item Osteopathy Clinical Teaching Questionnaire

Reliability estimate	Raw data	Polychoric correlation
Cronbach’s α	0.94	0.96
Guttman λ_6_	0.94	0.97
McDonald ω_hierarical_	0.86	0.87
McDonald ω_total_	0.95	0.97

With regard to ω, the ECV for the general factor (g) was 82% (raw data) and 84% (polychoric correlation), again consistent with the PSI. All items correlated substantially higher with the general factor (0.70 or higher) than with the two subfactors (0.4 or less) (Additional file 5) thereby supporting unidimensionality and the appropriateness of the calculation of a total score.

### Descriptive statistics

The descriptive statistics for the 12 items in the modified OCTQ are presented in Table 6. All items had a mean value greater than 4 (except for item 30 as it was rescored) and the median value for each item was 4 or 5. As the modified OCTQ was unidimensional, the calculation of a total score for the questionnaire is permissible (Table 6). The total possible score for the 12-item OCTQ is 59. The scoring structure and strata for each score is presented in Additional file 6.

**Table 6:** Descriptive statistics for the 12-item Osteopathy Clinical Teaching Questionnaire

Item	Mean	SD	Median	Minimum	Maximum	Standard error
2. Maintained a positive attitude towards me	4.46	0.89	5	1	5	0.04
5. Demonstrated humanistic attitudes in relating to patients (integrity, compassion and respect)	4.47	0.83	5	1	5	0.04
7. Showed genuine concern for my professional well-being	4.35	0.95	5	1	5	0.05
9. Has good communication skills	4.35	0.90	5	1	5	0.04
10. Is open to student questions and alternative approaches to patient management	4.34	0.93	5	1	5	0.05
12. Adjusted teaching to my needs (experience, competence, interest)	4.22	0.98	5	1	5	0.05
15. Promoted reflection on clinical practice	4.17	0.93	4	1	5	0.05
16. Emphasises a problem-solving approach rather than solutions	4.21	0.92	4	1	5	0.05
18. Asked questions to enhance my learning	4.27	0.91	5	1	5	0.05
20. Stimulates me to learn independently	4.23	0.89	4	1	5	0.04
23. Offered me suggestions for improvement when required	4.19	0.94	4	1	5	0.05
30. Demonstrated osteopathic, clinical examination and rehabilitation knowledge and skill(s)	3.54	0.72	4	1	4	0.04
Total score	50.83	8.40	53	17	59	0.42

## Discussion

The present study was designed to further evaluate the construct validity of the Osteopathy Clinical Teaching Questionnaire [16] using Rasch analysis. Tennant et al. [20] advocate the use of Rasch analysis in the development of unidimensional measures in the health sciences, and this type of analysis has previously been utilised to examine a questionnaire related to the clinical education of health profession students [22].

### Overall Rasch model fit

Data presented here suggest that the 12-item OCTQ satisfies the requirements of the Rasch model, that is, invariant measurement [45]. Rasch measurement “can be viewed as a psychometric model that can meet the requirements of IM [invariant measurement] when there is acceptable model-data fit” ([45], p.1375). Given an acceptable fit to the Rasch model was achieved after modifications, it is reasonable to conclude that these the 12-item OCTQ demonstrates the properties of invariant measurement. Further, the calculation of a total or summed score for the OCTQ is valid [46–48].

### Modifications to fit the Rasch model

#### Person misfit

Work by Curtis [49] has demonstrated that “…the inclusion of responses that underfit the Rasch measurement model, and that may reflect carelessness in responding, increase the standard errors of item estimates, reduce the range of item locations on the scale, and reduce the inter-threshold range within items” (p. 141). Further, Curtis’s work suggests that approximately 30% of respondents to an attitude survey may misfit (over- or under-fit) the Rasch model and require removal. This is consistent with the present study where 38% of the responses misfit the Rasch model. One example of why a response may be removed is that the participant circled all 5 s (strongly agree) for each item albeit that they are unlikely to strongly agree with each item [49]. By removing these responses in a systematic manner (Additional file 3), a fit to the Rasch model can be achieved.

#### Item misfit

Eighteen items did not fit the Rasch model at various stages during each iteration and were removed from the analysis in order to improve fit (Additional file 3). Both poor fit residuals and significant Bonferonni-adjusted chi-square values were observed initially for items 24 Identified areas needing improvement, and 25 Identified my strengths (Additional file 3). The wording of these items may have contributed to the misfit in that they are not specific to the feedback being provided by the clinical teacher as a part of their workplace learning, and rewording may improve their fit. Item 27 Promoted keeping of medical records in a way that is thorough, legible, efficient and organised demonstrated fit issues as observed on the ICC and the category probability curve, suggesting the item is measuring a construct that is inconsistent with the other OCTQ items. Respondents are being asked to make a judgment about the clinical teacher on a number of aspects of record keeping within the one item (i.e. thoroughness, organisation, efficiency), and this may be contributing to the misfit.

#### Item modification to achieve model fit

A strength of the Rasch measurement approach is the ability to rescore then reanalyse the fit of the item to the model [17, 50]. This approach can ensure that items that measure the latent construct are not removed when they can be modified to ensure a fit to the Rasch model. Respondents in the present study did not appear to be using the Strongly disagree and Disagree categories for item 30 Demonstrated osteopathic, clinical examination and rehabilitation knowledge and skill(s) – only 9 respondents (2%) used these categories. Although there was only a small number in each category, the responses to this item were inconsistent with that expected by the Rasch model. This item did not demonstrate misfit even though the thresholds were not being utilised in an ordered manner, a possibility identified by Hagquist [51]. Threshold disordering can introduce “…noise into the measurement” ([52], p.4733) and needs to be resolved either through removing the item or rescoring so that “…the threshold estimates located on the latent trait must appear in the same order as the manifest categories” ([51], p.514). It was appropriate to collapse the Strongly disagree and Disagree categories together as respondents are providing a negative response to the item regardless of which of the two categories they select. Item fit statistics improved with the rescoring of this item [18]. There are two important elements to note: 1) the item still has five categories on the questionnaire itself, it is only the scoring of the item during an analysis that changes (Additional file 6); and 2) it is not possible to collapse two categories together that do not make sense (i.e. collapse disagree with the neutral response category).

#### Differential item function

The presence of differential item function (DIF) violates the assumption of invariant measurement. Multiple items demonstrated DIF for institution, clinical teacher gender, and student gender in the present study. It is possible for one item demonstrating DIF to influence the fit of other items to the Rasch model [47, 51]. Item 14 Encouraged me to think demonstrated systematic differences for the same level of the underlying trait with regard to the three person-factors investigated. This systematic difference is termed uniform-DIF and given its presence across the three person-factors (institution, clinical teacher gender, and student gender). Pallant and Tennant [18] suggest such items are candidates for removal. None of the items in the present study demonstrated ‘artificial DIF’ [47]. No item in the 12-item OCTQ demonstrated either uniform or non-uniform DIF, meeting one of the assumptions of invariant measurement [47]. The analysis in the present study demonstrates that some items used in clinical teaching evaluations are affected by DIF and authors of subsequent evaluations should consider investigating the presence of DIF in the items contained within their questionnaire.

#### Person separation

The OCTQ PSI is acceptable and is sufficient to separate different levels of the underlying trait as perceived by the respondents [53]. A PSI of 0.85 or greater is reported to indicate a questionnaire is appropriate for decision-making about individuals (clinical teachers in the present study) [54] and the value of 0.827 in the present study suggests that the OCTQ could be used to make these decisions. This PSI value indicates there are likely four strata for the OCTQ [29] - this information could provide a degree of certainty in the decision making process. Such support is valuable, particularly where lower performing clinical teachers are identified for remediation by providing a statistical basis for the decision. Clinical teachers with a total OCTQ score of more than 32 are likely performing at an appropriate level as this value relates to the 4th strata (Additional file 6). Conversely, educators with a total OCTQ score of 25–32 (3rd strata) could be assisted with formal professional development activities or mentoring.

#### Binomial dimensionality testing & factor extraction

One of the strengths of the current study is the evidence-informed approach to the testing of the dimensionality of the OCTQ. Following the suggestions of Hagell [55, 56] and Engelhard Jr. [45], multiple methods were utilised to investigate the dimensionality of the OCTQ. The 95% confidence interval for the t-test between the items that loaded positively and negatively on the ‘Rasch factor’ contained the *p*-value of 0.05. Further, the number of factors to extract using the four extraction calculations was one, supporting the argument that the 12-item OCTQ is unidimensional.

### Reliability estimates

Further evidence for the unidimensionality of the OCTQ is provided through the ω and α values being well over the accepted value of 0.80. Although it has been suggested that the upper limit for α should be 0.90 [57] and values greater than this may indicate item redundancy [58], the fact that none of the 12 OCTQ items demonstrate local dependency (*r* < 0.20) suggests item redundancy is unlikely to be an issue.

McDonald’s ω_h_ [59] was also calculated for the OCTQ in order to investigate whether the items correlated more strongly with a general factor versus subfactors, and this was the case as evidenced by the path diagram at Additional file 5. ω_t_ is the estimate of the total reliability of a questionnaire including both the general factor and subfactors [44, 60]. The ω_t_ value in the present study is consistent with the Cronbach’s α value. Both the α and ω values suggest that over 94% of the total questionnaire score variance is due to all the factors in the model (both general and subfactors). ω_h_ on the other hand has been reported to be the most appropriate reliability estimation method [37] and represents the total questionnaire score variance due to the general factor or latent trait being measured [60]. In the present study over 85% of the total OCTQ score variance is due to the general factor as evidenced by ω_h_ values of 0.86 and 0.89 for the raw data and polychoric correlation respectively. These values are well above the 0.50 suggested by Revelle [40] and 0.70 suggested by Hermsen et al. [60], supporting the argument for unidimensionality of the OCTQ. Further support is provided by a large explained common variance (ECV) of 0.82 or 82% for the general factor using the raw data, and 84% for the polychoric correlation. The present study utilises multiple methods to provide evidence for the unidimensionality of a Rasch-derived measure.

### Targeting

The targeting of the thresholds of the OCTQ items covers a range of levels on the latent trait, particularly in the middle and lower ends of the scale. This targeting potentially allows the OCTQ to be used to identify clinical teachers who are perceived by the students to be performing suboptimally [61]. That said, respondents in the present study typically rated their clinical teachers highly, and this is consistent with reviews by Beckman et al. [62] and Fluit [7] on the validity evidence of clinical teaching evaluations. This potential ceiling effect is demonstrated by the mean person location value of 2.45. Whether this ceiling effect could be, or should be, reduced through modification of the response options is a matter for debate, as some of the clinical teachers in the present study could already be performing highly [61].

Support for the accuracy of the item and person location values is provided by the fact the initial 399 responses, and final 246 responses that demonstrated fit to the Rasch model, are greater than the sample size suggested by Linacre [63] and Pallant and Tennant [18]. Work by Linacre [63] suggests that a sample size of 243 will provide item and person location values that are accurate, regardless of the targeting of the scale.

### Developing the validity argument

The framework proposed by Kane [23] covers four stages: *scoring*, *generalisation*, *extrapolation* and *implications* and requires an initial definition of the latent construct under consideration. In the present study the latent construct is quality of clinical teaching provided in osteopathy on-campus, student-led clinics. Previous work has provided evidence to argue for the validity of the scores derived from version 2 of the OCTQ [16], particularly the *scoring* and *generalisation* arguments. The present study strengthens the *scoring* argument by evaluating the fit of the items and responses to the Rasch model, producing a questionnaire that meets the assumptions of invariant measurement. The unidimensionality of the OCTQ also provides a total score to estimate the latent construct thereby satisfying the requirements of a sufficient statistic, and provides further evidence for the *scoring* argument. The method by which the OCTQ is scored, along with the raw score-to-Rasch score conversion, is presented in Additional file 6. The total score (calculated by adding up each of the 12 items on the OCTQ) can be converted from an ordinal level raw score to a Rasch-derived interval level score that can be used in parametric statistical analyses. This data can then be used to evaluate the impact of faculty development activities, or track changes in clinical teacher performance over time.

The *generalisation* argument is also strengthened in that responses were collected from multiple students, rating multiple clinical teachers, at multiple institutions, in multiple countries. Initial development of version 2 of the OCTQ was focused on one institution, and the inclusion of institutions from New Zealand and the United Kingdom, in addition to Australia, progresses the *generalisation* argument. The evaluation of DIF, and subsequent removal of items that demonstrated this feature, provides evidence for the generalisation argument in that no item in the 12-item OCTQ produces different responses according to student gender, clinical teacher gender or institution.

Initial evidence for the *extrapolation* argument is also provided in the form of the OCTQ total score and item thresholds. The total score can be used to make judgements about the performance of a clinical teacher based on a students’ perception, and given the fit of the items to the Rasch model their thresholds can be used to differentiate between levels of clinical teacher performance at item level. These inferences are also supported by a PSI of over 0.80 for the OCTQ. Support for the *implications* argument is also presented in the form of the four statistically discrete strata that separate clinical teacher performance. By applying these strata, program administrators may be able to identify clinical teachers who would benefit from professional development activities or mentoring, as well as identifying those performing at the required level.

It is important to note that those elements described above are only parts of the validity argument, and not the argument as a whole. Further work is required to provide evidence for other aspects of Kane’s argument, particularly *generalisation* and *implications*, and this will be the subject of subsequent investigations using the 12-item OCTQ.

### Limitations of the study

Although the number of responses received was sufficient to undertake a Rasch analysis, the generalisability of the OCTQ is potentially limited to Australia, New Zealand and United Kingdom osteopathy teaching institutions. Further work would be required to argue for its use in teaching institutions in continental Europe, particularly around the validity of translations. There is also likely to be a degree of profession specificity in that the OCTQ has only been tested in the osteopathy profession. That said, it is possible that the questionnaire could be applied in other on-campus, student-led clinical teaching environments in professions such as chiropractic and podiatry, with only minor modifications. This assumption requires further testing. Item removal and modification was based both on the various fit statistics produced by RUMM, and the opinion of the author. Possible reasons for the removal of the 18 items from the OCTQ could have been explored through the use of a qualitative approach (asking students why they answered items in a particular way), either confirming the removal of the item or suggesting modifications for further testing. Further research is also required to investigate the relationship between the OCTQ and student age, and year level in their respective programs. Additionally, the influence of clinical educator demographics on OCTQ scores provides another avenue for investigation.

## Conclusion

The preceding analysis and discussion has provided further evidence to support the developing validity argument for the scores derived from the OCTQ, consistent with Kane’s approach to validity [23]. The present study has provided evidence to argue for the construct validity of a 12-item version of the OCTQ. The OCTQ is the first clinical teaching evaluation questionnaire to be developed using Rasch analysis during its initial stages, ensuring that it meets the assumptions of invariant measurement. Fit of the OCTQ items to the Rasch model and unidimensionality were achieved. Further evidence of unidimensionality was demonstrated through the omega hierarchical reliability estimate. The use of five response categories (*Strongly disagree* to *Strongly agree*) for 11 of the 12 items in the final version of the OCTQ is also supported by their fit to the Rasch model. Together this information supports the validity of using the total OCTQ score as a sufficient statistic representing the latent construct of clinical teaching quality in osteopathy.

Items were included in 4 of the 5 factors identified by Vaughan [16] in the initial development of the OCTQ. The learning environment, feedback, reflective practice and modelling factors all contributed items to the 12-item OCTQ however no item was drawn from the Patient Management factor.

The OCTQ has a number of uses. Firstly the questionnaire can be used as part of a quality assurance strategy in the clinical education component of a teaching program. Secondly, the results obtained from the OCTQ questionnaire can be used to inform faculty development or professional development activities to improve the clinical education experience for students and the educators, potentially improving patient care. Thirdly, the questionnaire has the potential to provide a focus for professional development activities. Finally, there is the potential for the questionnaire to be evaluated for use in allied health student-led clinics (or university clinics), including other non-United States osteopathy programs.

Further research is now required to evaluate the reliability of the 12-item OCTQ to strengthen the validity argument and determine how many evaluations need to be completed by students in order to obtain a reliable indication as to the quality of clinical teaching provided by a clinical teacher in osteopathy on-campus, student-led clinics.

## Additional files

Additional file 1: Osteopathy Clinical Teaching Questionnaire. (PDF 67 kb)

Additional file 2: Residual correlations. (PDF 35 kb)

Additional file 3: Steps in the Rasch analysis of the Osteopathy Clinical Teaching Questionnaire. (PDF 127 kb)

Additional file 4: Rationale for items retained or removed. (PDF 28 kb)

Additional file 5: McDonald's *omega* path diagrams for the 12-item Osteopathy Clinical Teaching Questionnaire. (PDF 117 kb)

Additional file 6: Scoring structure for the 12-item Osteopathy Clinical Teaching Questionnaire. (PDF 93 kb)
