# Supplementary material for: Exploring the measurement properties of the osteopathy clinical teaching questionnaire using Rasch analysis
Source: Chiropr Man Therap. 2018 May 3;26:13. doi: 10.1186/s12998-018-0182-2 (PMC5932865; doi:10.1186/s12998-018-0182-2)
Supplement: Supplementary file 1 — Osteopathy Clinical Teaching Questionnaire. (PDF 67 kb) [file 12998_2018_182_MOESM1_ESM.pdf]

Osteopathy Clinical Teaching Questionnaire

Using the following scale, please rate your Clinical Educator on the statements below:

5 – Strongly agree
4 – Agree
3 – Neither agree nor disagree
2 – Disagree
1 – Strongly disagree

Please indicate the gender of the Clinical Educator who is being rated:
☐ Male ☐ Female

Please indicate your gender:
☐ Male ☐ Female

This Clinical Educator…	Rating				
Treated me with respect	5	4	3	2	1
Maintained a positive attitude towards me	5	4	3	2	1
Fostered an environment of respect in which I felt comfortable participating	5	4	3	2	1
Established a good learning environment (approachable, focused, nonthreatening, professional and enthusiastic)	5	4	3	2	1
Demonstrated humanistic attitudes in relating to patients (integrity, compassion and respect)	5	4	3	2	1
Was approachable for discussion	5	4	3	2	1
Showed genuine concern for my professional well-being	5	4	3	2	1
Had reasonable expectations of students	5	4	3	2	1
Has good communication skills	5	4	3	2	1
Is open to student questions and alternative approaches to patient management	5	4	3	2	1
Gave me the opportunity to offer opinions on patient problems or treatment	5	4	3	2	1
Adjusted teaching to my needs (experience, competence, interest)	5	4	3	2	1
Is an effective clinical teacher	5	4	3	2	1
Encouraged me to think	5	4	3	2	1
Promoted reflection on clinical practice	5	4	3	2	1
Emphasises a problem-solving approach rather than solutions	5	4	3	2	1
Asked questions that promote learning (clarifies, probes, reflective questions etc.)	5	4	3	2	1
Asked questions to enhance my learning	5	4	3	2	1
Encouraged questions and active participation	5	4	3	2	1
Stimulates me to learn independently	5	4	3	2	1
Gave timely feedback to me	5	4	3	2	1
Gave me regular, useful feedback about my knowledge and performance	5	4	3	2	1
Offered me suggestions for improvement when required	5	4	3	2	1

Please turn over the page

Osteopathy Clinical Teaching Questionnaire

**This Clinical Educator…**	**Rating**
Identified areas needing improvement	5 4 3 2 1
Identified my strengths	5 4 3 2 1
Explained to me why I was correct or incorrect	5 4 3 2 1
Promoted keeping of medical records in a way that is thorough, legible, efficient and organised	5 4 3 2 1
Encouraged me to assume responsibility for patient care	5 4 3 2 1
Demonstrates knowledge of current medical and manual therapy literature	5 4 3 2 1
Demonstrated osteopathic, clinical examination and rehabilitation knowledge and skill(s)	5 4 3 2 1

Please rate your Clinical Educator on the following statements:

I would do more clinics with this Clinical Educator	5 4 3 2 1
I would recommend other students to work with this Clinical Educator	5 4 3 2 1

Rate the overall effectiveness of this Clinical Educator as an educator/supervisor:

☐ Poor ☐ Fair ☐ Good ☐ Very good ☐ Excellent

What have you enjoyed about working with this Clinical Educator?

____

____

____

____

____

____

Are there any areas where this Clinical Educator could improve or develop?

____

____

____

____

____

____
