# Supplementary material for: Exploring the measurement properties of the osteopathy clinical teaching questionnaire using Rasch analysis
Source: Chiropr Man Therap. 2018 May 3;26:13. doi: 10.1186/s12998-018-0182-2 (PMC5932865; doi:10.1186/s12998-018-0182-2)
Supplement: Supplementary file 2 — Residual correlations. (PDF 35 kb) [file 12998_2018_182_MOESM2_ESM.pdf]

Item	1	2	3	4	5	6	7	8	9	10	11	12	13	14	15	16	17	18	19	20	21	22	23	24	25	26	27	28	29	30
**1**	1																													
**2**	0.647	1																												
**3**	0.538	0.542	1																											
**4**	0.185	0.262	0.390	1																										
**5**	0.081	0.123	0.108	0.175	1																									
**6**	0.272	0.351	0.348	0.390	0.036	1																								
**7**	0.108	0.131	0.116	0.119	0.174	0.333	1																							
**8**	0.133	0.185	0.153	0.223	0.068	0.110	0.169	1																						
**9**	0.185	0.108	0.251	0.218	0.181	0.092	0.021	0.257	1																					
**10**	0.110	0.104	0.124	0.171	0.151	0.084	-0.067	0.148	0.218	1																				
**11**	0.074	0.104	0.081	0.061	0.044	0.062	-0.037	0.162	0.075	0.264	1																			
**12**	0.127	0.108	0.056	0.066	-0.043	0.015	-0.051	0.105	0.164	0.153	0.302	1																		
**13**	0.054	0.013	0.091	0.133	0.012	0.026	-0.112	-0.076	0.201	0.111	0.076	0.228	1																	
**14**	-0.178	-0.192	-0.196	-0.14	-0.128	-0.136	-0.161	-0.195	-0.121	-0.131	-0.085	-0.088	0.202	1																
**15**	-0.225	-0.174	-0.198	-0.099	-0.081	-0.052	0.080	-0.071	-0.171	-0.105	-0.111	-0.042	-0.030	0.224	1															
**16**	-0.171	-0.203	-0.186	-0.101	-0.099	-0.206	-0.051	-0.080	-0.034	-0.007	0.013	-0.064	0.079	0.295	0.277	1														
**17**	-0.131	-0.188	-0.199	-0.164	-0.094	-0.126	-0.124	-0.142	-0.113	-0.077	0.056	0.067	0.096	0.314	0.159	0.202	1													
**18**	-0.168	-0.168	-0.184	-0.134	-0.084	-0.172	-0.149	-0.19	-0.146	-0.084	-0.057	-0.079	0.082	0.332	0.092	0.206	0.558	1												
**19**	-0.074	-0.061	-0.108	-0.007	-0.042	-0.042	-0.001	-0.126	-0.116	-0.060	-0.036	-0.056	0.039	0.118	0.031	0.045	0.214	0.376	1											
**20**	-0.166	-0.069	-0.134	-0.087	0.059	-0.136	0.080	-0.114	-0.09	-0.157	-0.081	-0.044	-0.092	0.006	-0.006	0.042	-0.103	0.049	0.063	1										
**21**	-0.091	-0.097	-0.082	-0.206	-0.141	-0.134	-0.066	-0.132	-0.113	-0.203	-0.139	-0.165	-0.241	-0.205	-0.011	-0.041	-0.120	-0.141	-0.053	0.013	1									
**22**	-0.095	-0.186	-0.219	-0.235	-0.248	-0.098	-0.132	-0.151	-0.093	-0.182	-0.156	-0.166	-0.227	-0.150	-0.027	-0.099	-0.112	-0.116	-0.004	-0.020	0.541	1								
**23**	-0.086	-0.139	-0.158	-0.188	-0.169	-0.184	-0.124	-0.144	-0.112	-0.197	-0.178	-0.12	-0.220	-0.066	-0.042	-0.062	-0.095	-0.097	-0.166	0.007	0.178	0.408	1							
**24**	-0.296	-0.349	-0.297	-0.306	-0.208	-0.257	-0.157	-0.261	-0.254	-0.276	-0.299	-0.332	-0.216	0.035	-0.061	-0.053	-0.050	0.001	-0.124	-0.004	0.151	0.219	0.279	1						
**25**	-0.018	-0.085	-0.091	-0.147	-0.144	-0.095	-0.124	-0.056	-0.23	-0.109	-0.216	-0.122	-0.133	-0.219	-0.180	-0.250	-0.224	-0.216	-0.137	-0.081	0.082	0.112	0.125	0.331	1					
**26**	-0.141	-0.154	-0.102	-0.100	-0.148	-0.074	-0.153	-0.115	-0.141	-0.133	-0.163	-0.066	-0.076	-0.137	-0.141	-0.193	-0.110	-0.034	-0.053	-0.108	0.056	0.037	0.180	0.240	0.213	1				
**27**	-0.260	-0.255	-0.261	-0.233	-0.093	-0.184	-0.082	-0.091	-0.203	-0.198	-0.090	-0.096	-0.233	0.040	-0.043	-0.059	-0.082	-0.109	-0.163	0.031	0.017	0.055	-0.029	0.155	0.070	0.042	1			
**28**	-0.205	-0.169	-0.173	-0.247	0.049	-0.142	-0.016	-0.163	-0.175	-0.155	-0.114	-0.118	-0.155	0.123	-0.025	0.061	-0.086	-0.072	-0.088	0.070	0.093	-0.048	0.030	0.064	-0.052	0.044	0.255	1		
**29**	-0.177	-0.109	-0.128	-0.059	0.039	-0.156	-0.130	-0.125	-0.041	0.017	-0.053	-0.169	0.000	0.104	0.020	0.012	0.073	0.018	-0.006	-0.048	-0.138	-0.143	-0.099	0.056	-0.095	-0.081	-0.021	0.109	1	
**30**	-0.096	-0.121	-0.076	-0.044	0.008	-0.102	-0.138	-0.103	-0.033	0.015	0.058	-0.096	0.119	0.024	-0.098	-0.079	0.108	0.052	0.089	-0.054	-0.135	-0.156	-0.045	-0.007	-0.088	-0.040	-0.020	-0.026	0.396	1

Note: residual correlations >0.20 are highlighted.
