# Supplementary material for: Exploring the measurement properties of the osteopathy clinical teaching questionnaire using Rasch analysis
Source: Chiropr Man Therap. 2018 May 3;26:13. doi: 10.1186/s12998-018-0182-2 (PMC5932865; doi:10.1186/s12998-018-0182-2)
Supplement: Supplementary file 3 — Steps in the Rasch analysis of the Osteopathy Clinical Teaching Questionnaire. (PDF 127 kb) [file 12998_2018_182_MOESM3_ESM.pdf]

Iteration 1

Step	Description	Chi-square	PSI	Items	Person	Outcome
1	Initial analysis	407.42, p<0.0001	0.910	2.34	2.03	Item fit issues for 1, 3, 4, 17, 19, 24, 25, 27<br>Disordered thresholds for items 1, 9, 27, 30<br>DIF for institution (items 14, 27, 28), clinical educator gender (item 14), student gender (item 19)<br>122 misfitting persons<br>Local dependence multiple items
2	Rescore item 27	316.97, p<0.0001	0.928	2.13	1.11	Item fit issues for 17, 19, 24, 25, 27<br>Disordered thresholds for items 1, 30<br>DIF for institution (items 14, 27), clinical educator gender (item 14), student gender (item 19)<br>4 misfitting persons<br>Local dependence multiple items
3	Rescore item 30	316.87, p<0.0001	0.928	2.13	1.11	Item fit issues for 17, 19, 24, 25, 27<br>Disordered thresholds for items 1, 14<br>DIF for institution (items 14, 27), clinical educator gender (item 14), student gender (item 19)<br>4 misfitting persons<br>Local dependence multiple items
4	Delete item 27<br>(fit SD 6.497)	237.22, p<0.0001	0.926	1.83	1.07	Item fit issues for 19, 24, 25<br>Disordered thresholds for item 1<br>DIF for institution (items 14, 28), clinical educator gender (item 14), student gender (item 19)<br>3 misfitting persons<br>Local dependence multiple items
5	Delete item 24<br>(fit SD 3.985)	220.17, p<0.0001	0.924	1.83	1.02	Item fit issues for 19, 25<br>Disordered thresholds for item 1<br>DIF for institution (items 14, 28), clinical educator gender (item 14)<br>3 misfitting persons<br>Local dependence multiple items

6	Delete item 25<br>(fit SD 4.904)	186.89, p=0.002	0.916	1.74	1.01	Item fit issues for 19, 26<br>Disordered thresholds for item 1<br>DIF for institution (item 14), clinical educator gender (item 14)<br>7 misfitting persons<br>Local dependence multiple items
7	Delete 7 misfitting persons	186.21, p=0.002	0.922	1.73	1.01	Item fit issues for 19, 26<br>Disordered thresholds for items 1, 3<br>DIF for institution (item 14), clinical educator gender (item 14), student gender (item 19)<br>No misfitting persons<br>Local dependence multiple items
8	Delete item 14<br>(due to DIF)	184.17, p=0.001	0.920	1.75	1.02	Item fit issues for 19, 26<br>Disordered thresholds for items 1, 3<br>DIF for institution (item 28), student gender (item 19)<br>2 misfitting persons<br>Local dependence multiple items
9	Delete item 19<br>(due to DIF)	167.77, p=0.006	0.915	1.70	1.00	Item fit issues for 3, 26<br>Disordered thresholds for items 1, 3<br>No DIF<br>1 misfitting person<br>Local dependence multiple items
10	Delete item 3<br>(fit SD -2.566)	170.11, p=0.001	0.912	1.59	0.99	Item fit issue for 26<br>Disordered thresholds for item 1<br>DIF for institute (item 28)<br>1 misfitting person<br>Local dependence multiple items
11	Delete item 26<br>(fit SD 2.974)	138.57, p=0.066	0.903	1.57	0.97	Item fit issues<br>Disordered thresholds for item 1<br>No DIF<br>5 misfitting persons<br>Local dependence multiple items

12	Delete 5 misfitting persons	137.08, p=0.078	0.907	1.56	0.95	No item fit issues<br>Disordered thresholds for item 1<br>No DIF<br>No misfitting persons<br>Local dependence multiple items
13	Delete item 1 (unable to resolve disordering)	139.43, p=0.030	0.904	1.50	0.95	No item fit issues<br>No disordered thresholds<br>No DIF<br>2 misfitting persons<br>Local dependence multiple items
14	Delete 2 misfitting persons	135.76, p=0.048	0.904	1.52	0.93	No item fit issues<br>No disordered thresholds<br>DIF for institute (item 28)<br>No misfitting persons<br>Local dependence multiple items
15	Delete item 28 (due to DIF)	133.23, p=0.030	0.904	1.58	0.92	No item fit issues<br>No disordered thresholds<br>No DIF<br>4 misfitting persons<br>Local dependence multiple items
16	Delete 4 misfitting persons	129.61, p=0.051	0.901	1.55	0.90	No item fit issues<br>No disordered thresholds<br>No DIF<br>No misfitting persons<br>Local dependence multiple items
17	Delete item 17 (after subtest of items 17/18 due to r=0.550)	124.98, p=0.046	0.894	1.50	0.81	Item fit issues for 4, 9<br>No disordered thresholds<br>No DIF<br>No misfitting persons<br>Local dependence multiple items
18	Delete item 21 (after subtest of	134.01, p=0.005	0.888	1.62	0.85	Item fit issues for 4, 9<br>No disordered thresholds

	items 21/22 due to r=0.540)					No DIF<br>2 misfitting persons<br>Local dependence multiple items
19	Delete 2 misfitting persons	134.01, p=0.005	0.890	1.62	0.85	Item fit issues for 4, 9<br>No disordered thresholds<br>No DIF<br>2 misfitting persons<br>Local dependence multiple items
20	Delete item 9<br>(fit SD -2.718)	111.62, p=0.061	0.881	1.49	0.84	Item fit issues for 4<br>No disordered thresholds<br>No DIF<br>1 misfitting person<br>Local dependence multiple items
21	Delete misfitting person	111.62, p=0.061	0.882	1.49	0.84	Item fit issues for 4<br>No disordered thresholds<br>No DIF<br>No misfitting persons<br>Local dependence multiple items
22	Delete item 4<br>(fit SD -2.697)	102.94, p=0.090	0.874	1.35	0.84	No item fit issues<br>No disordered thresholds<br>No DIF<br>No misfitting persons<br>Local dependence multiple items
23	Delete item 6 (after subtest of items 6/7 r=0.325, and 2/6 r=0.276)	97.44, p=0.089	0.868	1.35	0.83	No item fit issues<br>No disordered thresholds<br>No DIF<br>2 misfitting persons<br>Local dependence multiple items
24	Delete 2 misfitting persons	97.19, p=0.092	0.868	1.34	0.82	No item fit issues<br>No disordered thresholds<br>No DIF<br>No misfitting persons

						Local dependence multiple items
25	Delete item 22 (after subtest of items 22/23 r=0.410)	88.535, p=0.135	0.854	1.50	0.85	No item fit issues<br>No disordered thresholds<br>No DIF<br>7 misfitting persons<br>Local dependence multiple items
26	Delete 7 misfitting persons	86.89, p=0.163	0.843	1.44	0.80	No item fit issues<br>No disordered thresholds<br>DIF for institution (item 13)<br>No misfitting persons<br>Local dependence multiple items
27	Delete item 13 (due to DIF)	91.17, p=0.045	0.830	1.29	0.83	No item fit issues<br>No disordered thresholds<br>No DIF<br>1 misfitting persons<br>Local dependence multiple items
28	Delete item 12 (after subtest of items 11/12 r=0.220)	66.47, p=0.425	0.809	1.20	0.82	No item fit issues<br>No disordered thresholds<br>No DIF<br>1 misfitting persons<br>Local dependence multiple items
29	Delete item 29 (after subtest of items 29/30 r=0.338)	66.06, p=0.275	0.800	1.20	0.83	No item fit issues<br>No disordered thresholds<br>No DIF<br>3 misfitting persons<br>No local dependence
30	Delete 3 misfitting persons	66.06, p=0.275	0.802	1.20	0.83	p-value range 0.061<br>Lower CI 0.031-0.038<br>Upper CI 0.091-0.101

Iteration 2

Step	Description	Chi-square	PSI	Items	Person	Outcome
1	Initial analysis	407.42, p<0.0001	0.910	2.34	2.03	Item fit issues for 1, 3, 4, 17, 19, 24, 25, 27<br>Disordered thresholds for items 1, 9, 27, 30<br>DIF for institution (items 14, 27, 28), clinical educator gender (item 14), student gender (item 19)<br>122 misfitting persons<br>Local dependence multiple items
2	Delete item 27 (fit SD 6.138)	237.36, p<0.0001	0.926	1.83	1.07	Item fit issues for 19, 24, 25<br>Disordered thresholds for items 1, 30<br>DIF for institution (items 14, 28), clinical educator gender (item 14), student gender (item 19)<br>3 misfitting persons<br>Local dependence multiple items
3	Delete item 19 (due to DIF and fit SD -3.229)	236.59, p<0.0001	0.922	1.74	1.05	Item fit issues for 24, 25<br>Disordered thresholds for items 1, 14, 30<br>DIF for institution (items 14, 28), clinical educator gender (item 14)<br>3 misfitting persons<br>Local dependence multiple items
4	Delete 3 misfitting persons	239.02, p<0.0001	0.921	1.68	1.03	Item fit issues for 24, 25<br>Disordered thresholds for items 1, 21, 30<br>DIF for institution (items 14, 28), clinical educator gender (item 14)<br>No misfitting persons<br>Local dependence multiple items
5	Rescore item 21	239.02, p<0.0001	0.921	1.68	1.03	Item fit issues for 24, 25<br>Disordered thresholds for items 1, 30<br>DIF for institution (items 14, 28), clinical educator gender (item 14)<br>No misfitting persons<br>Local dependence multiple items
6	Rescore item 30	239.02, p<0.0001	0.921	1.68	1.03	Item fit issues for 24, 25

						Disordered thresholds for item 1<br>DIF for institution (items 14, 26), clinical educator gender (item 14)<br>No misfitting persons<br>Local dependence multiple items
7	Delete item 1<br>(unable to resolve disordering)	218.47, p<0.0001	0.919	1.63	1.03	Item fit issues for 24, 25<br>No disordered thresholds<br>DIF for institution (items 14, 28), clinical educator gender (item 14)<br>1 misfitting person<br>Local dependence multiple items
8	Delete item 24<br>(fit SD 3.720)	204.69, p<0.0001	0.916	1.62	0.98	Item fit issue for 25<br>No disordered thresholds<br>DIF for institution (items 14), clinical educator gender (item 14)<br>No misfitting persons<br>Local dependence multiple items
9	Delete item 25<br>(fit SD 4.164)	155.73, p=0.032	0.908	1.56	0.98	Item fit issue for 26<br>No disordered thresholds<br>DIF for institution (items 14), clinical educator gender (item 14)<br>5 misfitting persons<br>Local dependence multiple items
10	Delete 5 misfitting persons	155.73, p=0.032	0.913	1.56	0.98	Item fit issue for 26<br>No disordered thresholds<br>DIF for institution (items 14), clinical educator gender (item 14)<br>No misfitting persons<br>Local dependence multiple items
11	Delete item 26	148.91, p=0.037	0.905	1.53	0.95	No item fit issues<br>No disordered thresholds<br>DIF for institution (items 14), clinical educator gender (item 14)<br>6 misfitting persons<br>Local dependence multiple items
12	Delete 6 misfitting persons	139.61, p=0.106	0.909	1.54	0.93	No item fit issues<br>No disordered thresholds<br>DIF for institution (items 14), clinical educator gender (item 14)

						No misfitting persons<br>Local dependence multiple items
13	Delete item 14	133.91, p=0.109	0.906	1.56	0.93	No item fit issues<br>No disordered thresholds<br>No DIF<br>No misfitting persons<br>Local dependence multiple items
14	Delete item 17 (after subtest 17/18, r=0.567)	129.22, p=0.101	0.900	1.55	0.91	Item fit issue for 4<br>No disordered thresholds<br>DIF for institution (item 13)<br>No misfitting persons<br>Local dependence multiple items
15	Delete item 3 (after subtest 2/3, r=0.487)	117.81, p=0.185	0.896	1.42	0.91	No item fit issues<br>No disordered thresholds<br>DIF for institution (item 13)<br>No misfitting persons<br>Local dependence multiple items
16	Delete item 13	106.72, p=0.304	0.890	1.31	0.91	No item fit issues<br>No disordered thresholds<br>No DIF<br>No misfitting persons<br>Local dependence multiple items
17	Delete item 6 (after subtest 2/6 r=0.325 & 4/6 r=0.343	103.15, p=0.266	0.886	1.23	0.90	No item fit issues<br>No disordered thresholds<br>No DIF<br>No misfitting persons<br>Local dependence multiple items
18	Delete item 11 (after subtest 10/11 r=0.202 & 11/12 r=0.251)	97.61, p=0.273	0.880	1.14	0.90	No item fit issues<br>No disordered thresholds<br>DIF for institution (item 28)<br>No misfitting persons<br>Local dependence multiple items

19	Delete item 29 (after subtest 29/30 r=0.374)	87.78, p=0.396	0.876	1.14	0.88	No item fit issues<br>No disordered thresholds<br>DIF for institution (item 28)<br>2 misfitting persons<br>Local dependence multiple items
20	Delete 2 persons	87.78, p=0.396	0.878	1.14	0.88	No item fit issues<br>No disordered thresholds<br>DIF for institution (item 28)<br>No misfitting persons<br>Local dependence multiple items
21	Delete item 28	88.62, p=0.238	0.873	1.15	0.87	No item fit issues<br>No disordered thresholds<br>No DIF<br>2 misfitting persons<br>Local dependence multiple items
22	Delete 2 persons	88.62, p=0.238	0.874	1.15	0.87	No item fit issues<br>No disordered thresholds<br>No DIF<br>No misfitting persons<br>Local dependence multiple items
23	Delete item 21 (after subtest 21/22 r=0.511)	96.36, p=0.04	0.864	1.33	0.84	No item fit issues<br>No disordered thresholds<br>No DIF<br>2 misfitting persons<br>Local dependence multiple items
24	Delete 2 persons	96.36, p=0.04	0.866	1.33	0.84	No item fit issues<br>No disordered thresholds<br>No DIF<br>No misfitting persons<br>Local dependence multiple items
25	Delete item 8 (after subtest 8/9	73.60, p=0.360	0.859	1.17	0.85	No item fit issues<br>No disordered thresholds

	r=0.202)					No DIF<br>1 misfitting person<br>Local dependence multiple items
26	Delete item 4 (after subtest 2/4 r=0.207)	62.40, p=0.568	0.846	1.05	0.85	No item fit issues<br>No disordered thresholds<br>No DIF<br>3 misfitting persons<br>Local dependence items 22 & 23
27	Delete 3 persons	62.63, p=0.560	0.844	1.05	0.84	No item fit issues<br>No disordered thresholds<br>No DIF<br>No misfitting persons<br>Local dependence items 22 & 23
28	Delete item 22 (after subtest 22/23 r=0.379)	70.78, p=0.160	0.826	1.24	0.85	No item fit issues<br>No disordered thresholds<br>No DIF<br>8 misfitting persons<br>No local dependence
29	Delete 8 persons	65.26, p=0.298	0.827	1.18	0.82	p-value range 0.02<br>Lower CI 0.002-0.008<br>Upper CI 0.037-0.049
