# Supplementary material for: Exploring the measurement properties of the osteopathy clinical teaching questionnaire using Rasch analysis
Source: Chiropr Man Therap. 2018 May 3;26:13. doi: 10.1186/s12998-018-0182-2 (PMC5932865; doi:10.1186/s12998-018-0182-2)
Supplement: Supplementary file 4 — Rationale for items retained or removed. (PDF 28 kb) [file 12998_2018_182_MOESM4_ESM.pdf]

Item	Factor*	Description
1. Treated me with respect	LE	Disordered thresholds unable to be resolved through rescoring
2. Maintained a positive attitude towards me	LE	Retained
3. Fostered an environment of respect in which I felt comfortable participating	LE	Removed due to local dependence with item 2
4. Established a good learning environment (approachable, focused, nonthreatening, professional and enthusiastic)	LE	Removed due to local dependence with item 2
5. Demonstrated humanistic attitudes in relating to patients (integrity, compassion and respect)	LE	Retained
6. Was approachable for discussion	LE	Removed due to local dependence with item 2
7. Showed genuine concern for my professional well-being	LE	Retained
8. Had reasonable expectations of students	LE	Removed due to local dependence with item 9
9. Has good communication skills	LE	Retained

10. Is open to student questions and alternative approaches to patient management	LE	Retained
11. Gave me the opportunity to offer opinions on patient problems or treatment	LE	Removed due to local dependence with items 10 and 12
12. Adjusted teaching to my needs (experience, competence, interest)	LE	Retained
13. Is an effective clinical teacher	LE	Demonstrated DIF for university
14. Encouraged me to think	RP	Demonstrated DIF for both university & clinical educator gender
15. Promoted reflection on clinical practice	RP	Retained
16. Emphasises a problem-solving approach rather than solutions	RP	Retained
17. Asked questions that promote learning (clarifies, probes, reflective questions etc.)	RP	Removed due to local dependence with item 18
18. Asked questions to enhance my learning	RP	Retained
19. Encouraged questions and active participation	RP	Fit residual SD = -3.229

20. Stimulates me to learn independently	RP	Retained
21. Gave timely feedback to me	FB	Removed due to local dependence with item 22
22. Gave me regular, useful feedback about my knowledge and performance	FB	Removed due to local dependence with item 23
23. Offered me suggestions for improvement when required	FB	Retained
24. Identified areas needing improvement	FB	Fit residual SD = 3.720, χ^2=36.96, p<0.001
25. Identified my strengths	FB	Fit residual SD = 4.164, χ^2=36.56, p<0.001
26. Explained to me why I was correct or incorrect	FB	Fit residual SD = 3.179
27. Promoted keeping of medical records in a way that is thorough, legible, efficient and organised	PM	Fit residual SD = 6.938, χ^2=85.27, p<0.001
28. Encouraged me to assume responsibility for patient care	PM	Demonstrated DIF for university
29. Demonstrates knowledge of current medical and manual therapy literature	MD	Removed due to local dependence with item 30

30. Demonstrated osteopathic, clinical examination and rehabilitation knowledge and skill(s)	MD	Retained but rescored

*Factors identified in the paper by Vaughan (2015): LE – learning environment; RP – reflective practice; FB – feedback; PM – patient management; MD – modelling.
