# Supplementary material for: Exploring the measurement properties of the osteopathy clinical teaching questionnaire using Rasch analysis
Source: Chiropr Man Therap. 2018 May 3;26:13. doi: 10.1186/s12998-018-0182-2 (PMC5932865; doi:10.1186/s12998-018-0182-2)
Supplement: Supplementary file 5 — McDonald's omega path diagrams for the 12-item Osteopathy Clinical Teaching Questionnaire. (PDF 117 kb) [file 12998_2018_182_MOESM5_ESM.pdf]

Omega path diagram (raw data)	Omega path diagram (polychoric correlation)
see below	see below

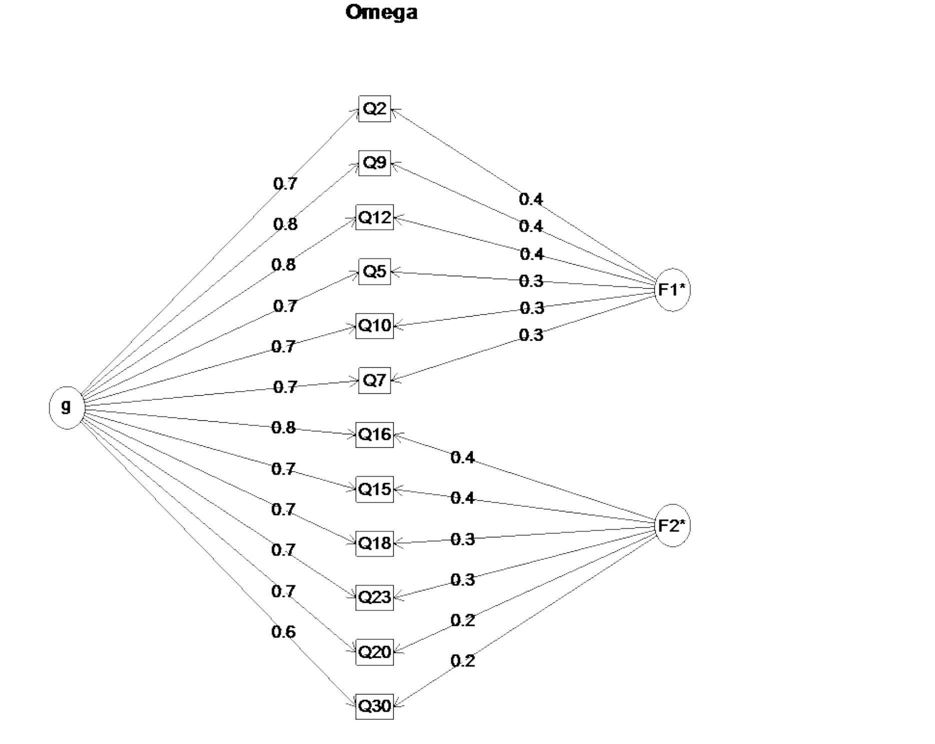

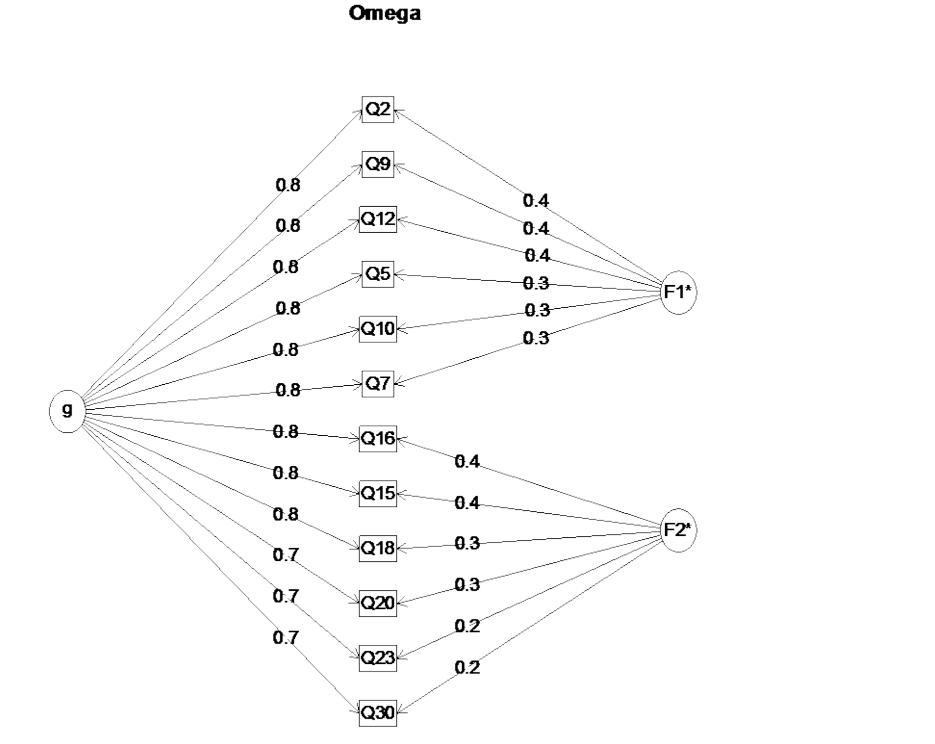

2. Maintained a positive attitude towards me
5. Demonstrated humanistic attitudes in relating to patients (integrity, compassion and respect)
7. Showed genuine concern for my professional well-being
9. Has good communication skills
10. Is open to student questions and alternative approaches to patient management
12. Adjusted teaching to my needs (experience, competence, interest)
15. Promoted reflection on clinical practice
16. Emphasises a problem-solving approach rather than solutions
18. Asked questions to enhance my learning
20. Stimulates me to learn independently
23. Offered me suggestions for improvement when required
30. Demonstrated osteopathic, clinical examination and rehabilitation knowledge and skill(s)
