# Supplementary material for: Exploring the measurement properties of the osteopathy clinical teaching questionnaire using Rasch analysis
Source: Chiropr Man Therap. 2018 May 3;26:13. doi: 10.1186/s12998-018-0182-2 (PMC5932865; doi:10.1186/s12998-018-0182-2)
Supplement: Supplementary file 6 — Scoring structure for the 12-item Osteopathy Clinical Teaching Questionnaire. (PDF 93 kb) [file 12998_2018_182_MOESM6_ESM.pdf]

Scoring structure for the 12-item Osteopathy Clinical Teaching Questionnaire.

OCTQ
OSTEOPATHY CLINICAL TEACHING QUESTIONNAIRE

Item	Strongly disagree	Disagree	Neither agree nor disagree	Agree	Strongly agree
1. Maintained a positive attitude towards me	1	2	3	4	5
2. Demonstrated humanistic attitudes in relating to patients (integrity, compassion and respect)	1	2	3	4	5
3. Showed genuine concern for my professional well-being	1	2	3	4	5
4. Has good communication skills	1	2	3	4	5
5. Is open to student questions and alternative approaches to patient management	1	2	3	4	5
6. Adjusted teaching to my needs (experience, competence, interest)	1	2	3	4	5
7. Promoted reflection on clinical practice	1	2	3	4	5
8. Emphasises a problem-solving approach rather than solutions	1	2	3	4	5
9. Asked questions to enhance my learning	1	2	3	4	5
10. Stimulates me to learn independently	1	2	3	4	5
11. Offered me suggestions for improvement when required	1	2	3	4	5
12. Demonstrated osteopathic, clinical examination and rehabilitation knowledge and skill(s)	1	1	2	3	4

Raw score to Rasch score conversion for the 12-item Osteopathy Clinical Teaching Questionnaire.

OCTQ
OSTEOPATHY CLINICAL TEACHING QUESTIONNAIRE

Total score	Rasch score	Strata
12	0	1
13	1	1
14	1	1
15	2	1
16	3	1
17	4	1
18	6	1
19	8	2
20	10	2
21	13	2
22	15	2
23	18	2
24	21	2
25	24	3
26	27	3
27	30	3
28	33	3
29	36	3
30	39	3
31	42	3
32	45	3
33	48	4

Total score	Rasch score	Strata
34	51	4
35	53	4
36	56	4
37	59	4
38	61	4
39	64	4
40	67	4
41	69	4
42	72	4
43	74	4
44	76	4
45	78	4
46	80	4
47	82	4
48	84	4
49	86	4
50	88	4
51	90	4
52	91	4
53	93	4
54	94	4
55	96	4

Total score	Rasch score	Strata
56	97	4
57	98	4
58	99	4
59	100	4
